# Regional patient transfer patterns matter for the spread of hospital-acquired pathogens

**DOI:** 10.1038/s41598-023-50873-z

**Published:** 2024-01-09

**Authors:** Hanjue Xia, Johannes Horn, Monika J. Piotrowska, Konrad Sakowski, André Karch, Mirjam Kretzschmar, Rafael Mikolajczyk

**Affiliations:** 1 Institute for Medical Epidemiology, Biometrics and Informatics (IMEBI), Interdisciplinary Centre for Health Sciences, Medical School of the Martin Luther University Halle-Wittenberg, 06108 Halle, Saale Germany; 2 Institute of Applied Mathematics and Mechanics, University of Warsaw, 02-097 Warsaw, Poland; 3 Institute for Epidemiology and Social Medicine, University of Münster, 48149 Münster, Germany; 4 Julius Centre for Health Sciences and Primary Care, University Medical Centre Utrecht, Utrecht University, 3584 CG Utrecht, The Netherlands

**Keywords:** Computational biology and bioinformatics, Computational models

## Abstract

Pathogens typically responsible for hospital-acquired infections (HAIs) constitute a major threat to healthcare systems worldwide. They spread via hospital (or hospital-community) networks by readmissions or patient transfers. Therefore, knowledge of these networks is essential to develop and test strategies to mitigate and control the HAI spread. Until now, no methods for comparing healthcare networks across different systems were proposed. Based on healthcare insurance data from four German federal states (Bavaria, Lower Saxony, Saxony and Thuringia), we constructed hospital networks and compared them in a systematic approach regarding population, hospital characteristics, and patient transfer patterns. Direct patient transfers between hospitals had only a limited impact on HAI spread. Whereas, with low colonization clearance rates, readmissions to the same hospitals posed the biggest transmission risk of all inter-hospital transfers. We then generated hospital-community networks, in which patients either stay in communities or in hospitals. We found that network characteristics affect the final prevalence and the time to reach it. However, depending on the characteristics of the pathogen (colonization clearance rate and transmission rate or even the relationship between transmission rate in hospitals and in the community), the studied networks performed differently. The differences were not large, but justify further studies.

## Introduction

Healthcare-associated infections (HAIs) threaten individual patients’ health as well as healthcare systems worldwide. Each year 3.1–4.6 million people acquire a HAI in acute care hospitals in European Union (EU) countries, Iceland, Norway, and the United Kingdom^1^. The emergence of HAIs not only increases morbidity and mortality, but also triggers psychological problems for patients as well as a financial burden for the healthcare system. Each year, more than 90,000 people die in EU countries, including Iceland, Norway, and the United Kingdom due to the six most common HAIs^2^. HAIs are the single most deadly and costly adverse event, representing up to $6\%$ of public hospital budgets^3^, and nowadays HAI spreading is a universal public health threat at local and national scales.

In recent years, English, Dutch, as well as French national medical registration datasets have been used to construct “healthcare networks” to provide insights into patient transfer management, hospital infection prevention and control. In these networks, nodes represented hospitals and edges between pairs of nodes represented patient transfers between the linked hospitals. The networks were used for simulating the spread of HAIs^4–18^, evaluating epidemic risks^5,6,10–18^, and recommending control strategies^5,6,10–18^. Patients’ and hospitals’ characteristics, as well as patient transfer patterns, may differ in different regions of a country, leading to the different spread of pathogens^11,14^. These regional variations were not considered yet. Some recent studies focused on incomplete network data^4^ and modeling the epidemic spread^4,5,11–18^. Nevertheless, a comparison of healthcare networks from different regions or countries with heterogeneous patients’ and hospitals’ characteristics as well as patient transfer patterns, has not been done yet. Here, we fill this gap and present an analysis of different regions of Germany which with its federal structure might have more regional diversity, but our findings can be still informative for other settings.

## Results

The results address several aspects of the conducted research. First, the general properties of the datasets after the scale-up procedure are discussed. Then, properties of the network models of the regional healthcare networks, derived from the up-scaled datasets are considered. Finally simulation of dynamics of Klebsiella (as a model pathogen for HAI) spread is presented, which is the main result of this study.

### Federal scaled-up datasets

In the scaled-up datasets, the largest numbers of hospitals and hospitalized patients were in Bavaria, reflecting the larger population. The average LOSs (length of stay) in hospitals were 9 days or longer, which perfectly matched the Germany-wide average LOS^19^. In Saxony and Thuringia, hospitals were on average larger and had longer patient stays than in the other two federal states. In contrast, the average numbers of hospitalizations per patient were close to each other (cf. Table 1).

**Table 1:** Basic characteristics of hospitals and patient stays in three federal states for the scaled-up datasets.

Federal States	Bavaria	Lower Saxony	Saxony and Thuringia**
Time span (years)	2010–2015	2010–2015	2011–2016
Number of hospitals	357	211	126
Number of patients with at least one hospitalization during six years (CI*)	7590768 ([7396910, 7784625])	4287973 ([4204486, 4371461])	3339086 ([3272147, 3406025])
Average number of occupied beds per hospital per day (CI*)	241.3 ([234.8, 247.8])	223.2 ([218.7, 227.7])	316.7 ([310.0, 323.4])
Average length (days) of hospital stays (CI*)	9.1 ([9.1, 9.1])	9.1 ([9.1, 9.1])	9.7 ([9.7, 9.7])
Average time (days) between two successive hospital stays (CI*)	255.6 ([255.4, 255.8])	262.0 ([261.8, 262.1])	242.9 ([242.8, 243.0])
Average number of hospitalizations per patient during six years (CI*)	2.7 ([2.7, 2.7])	2.7 ([2.6, 2.7])	2.7 ([2.7, 2.7])

$^{*}$We generated 100 different federal scaled-up dataset samples and calculated the statistics above for each federal scaled-up dataset. Then we computed empirical $95\%$ confidence intervals (CIs) for each statistic.

$$^{**}$$While we have data from two federal states provided by the AOK Plus, we cannot separate them in the dataset.

To provide further information about the hospital and patient characteristics, we also presented the distribution of hospital- and community-node sizes in Supplemental Fig. S5, in hospital groups defined by size in Supplemental Fig. S7, of admissions in different age groups in Supplemental Fig.S8, of LOSs in Supplemental Fig. S9, of the number of hospitalizations in Supplemental Fig. S10, as well as dependencies of corresponding community-node sizes on hospital-node sizes in Supplemental Fig. S6.

We found that Lower Saxony had the largest proportion of medium size hospitals, and the proportion of large hospitals was biggest in Saxony and Thuringia (see Supplemental Figs. S5 and S7). The sizes of community nodes were positively correlated to the sizes of hospital nodes (see Supplemental Fig. S5). The community nodes were much larger than the hospital nodes and the ratio was higher than 80:1 in the studied federal states. The patient age distributions were very similar in Bavaria and Lower Saxony, whereas Saxony and Thuringia had larger proportions of older patients (see Fig. S8). Consequently, the proportion of long LOSs in Saxony and Thuringia was larger than in the other two federal states (see Fig. S9). However, the distributions of the number of hospitalizations per person did not show much difference across the federal states and nearly half of the patients (around $45\%$) had only been hospitalized once in six years (see Supplemental Fig.  S10). Fewer patients had been hospitalized twice (around $22.5\%$), three times (around $12.5\%$), four times (around $7.5\%$) or more often (around $12.5\%$).

### Properties of the hospital networks

**Figure 1:**
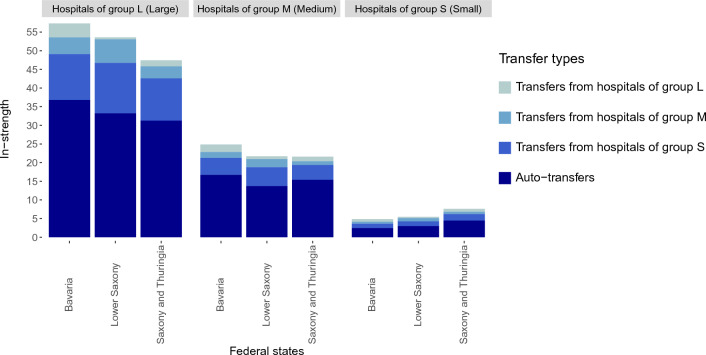
In-strengths for hospitals originating from auto-transfers and from transfers from different hospitals, categorized by hospital sizes *L* (large), *M* (medium), *S* (small), by federal states.

First, we constructed the hospital network based on the original parameter settings for ESBL-Klebsiella, as introduced in “Network construction” of “Methods”. Through the hospital network, we can directly identify and assess the risk of transmission posed by the transfer of patients between hospitals. The effective edge weights are distributed similarly in these four federal states (see Supplemental Fig. S11), and the effective weights of more than $90\%$ of edges were less than 0.5 patients per day. The distributions of in- and out-strengths were nearly the same, and both of them showed that the larger the hospital sizes, the higher the hospital network strengths (see Fig. 1 and Supplemental Fig. S12). Because for most hospitals the largest portion of the strengths was related to auto-transfers, auto-transfers triggered the largest risk of epidemic spread (see Fig. 1 and Supplemental Fig.  S12). Generally, there were $62.3\%$ auto-transfers in Saxony and Thuringia in total, whereas only $57.1\%$ in total in Bavaria and Lower Saxony. Besides auto-transfers, hospital strengths originating from small hospitals were also relatively strong, whereas the strengths originating from large hospitals were the weakest (Fig. 1 and Supplemental Fig. S12).

**Figure 2:**
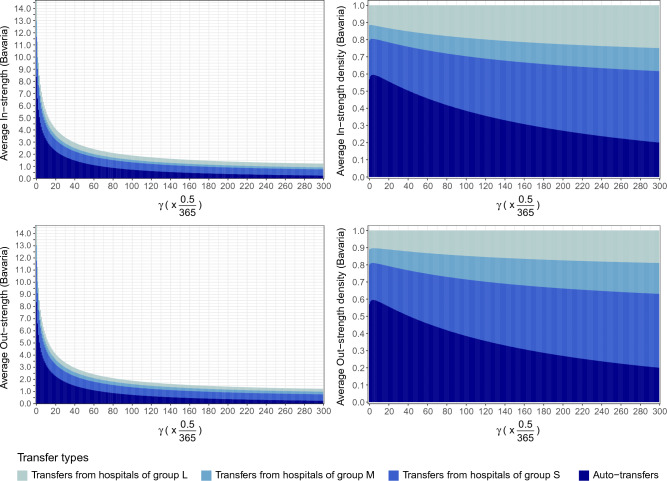
Distributions and density function of average in- and out-strengths of hospitals originating from auto-transfers and from hospitals categorized by hospital sizes (*L*, *M*, *S*) for varying colonization clearing rates $\gamma$ in Bavaria.

We also used the same basic hospital networks with varying the colonization clearing rate and thereby the effective edge weights, which led to different distributions of hospital in- and out-strengths. As shown in Fig. 2, Supplemental Figs. S12 and S13, we found that the average strengths originating from auto-transfers decreased more sharply with increasing colonization clearing rate than the strengths of other types of transfers.

### Spread of HAI within the hospital-community network

**Figure 3:**
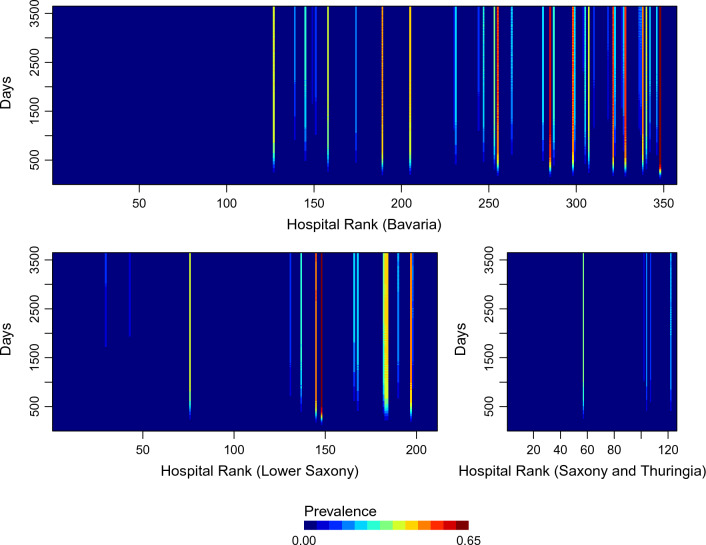
Prevalence at admission. The x-axis represents hospitals, ranked from largest to smallest (left to right) based on the average number of hospital beds that were occupied per day.

In the baseline scenario (i.e. no community node transmission), the time for reaching the final states in individual hospital and community nodes was highly heterogeneous (Fig. 3, Supplemental Fig. S15). In Saxony and Thuringia, $80\%$ of the nodes reached the final state within 3000 days. However, in Bavaria and Lower Saxony, the hospitals took longer to reach their final states. The final prevalence in most hospitals was very low (ranging from 0 to 0.004) (see Fig. 3). High final prevalence (ranging from 0.3 to 0.65) appeared only in some of the small hospitals which were ranked higher than 60 in Bavaria and Lower Saxony as well as higher than 50 in Saxony and Thuringia (see Table 2; Fig. 3). To check whether hospital prevalence is correlated with hospital size, we calculated the Pearson correlation. The correlation was $-0.15$ in Bavaria, $-0.13$ in Lower Saxony and $-0.08$ in Saxony and Thuringia. We also calculated the Pearson correlation between the hospital prevalence and the LOS, resulting in 0.91 in Bavaria and Lower Saxony, and 0.73 in Saxony and Thuringia. This suggests that the higher prevalence may result from longer LOSs. Moreover, the prevalence fluctuated on different days of the week, likely due to the daily variation in patient transfer probabilities. In comparison to the healthcare networks in Bavaria and Lower Saxony, the hospital node prevalences in Saxony and Thuringia were lower.

**Figure 4:**
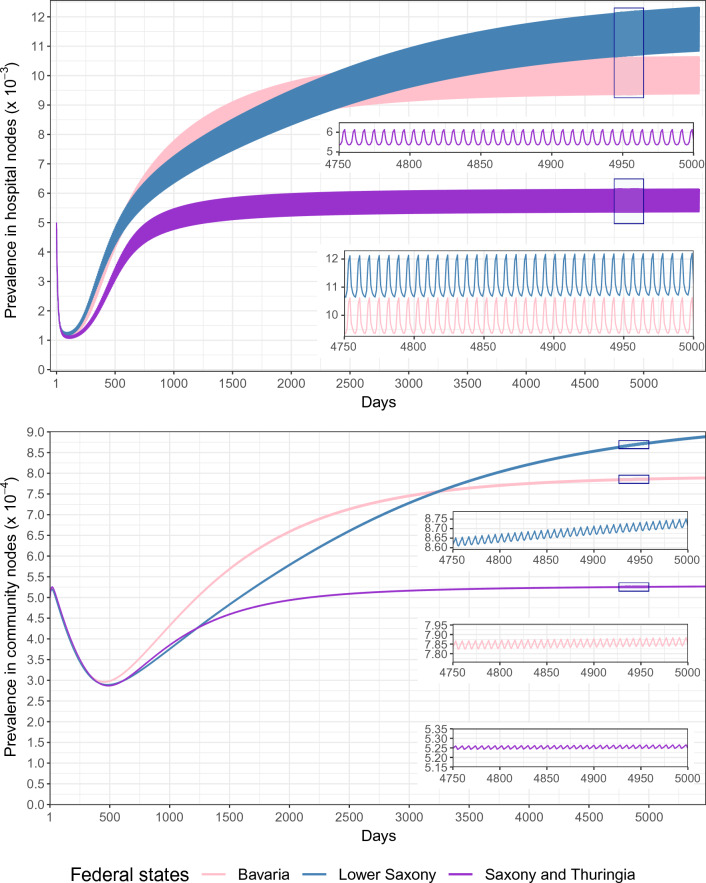
Prevalence at admission to hospitals, evolving over time since the introduction of the pathogen, by the federal state; sections are enlarged to illustrate the weekly prevalence variations.

The prevalence of ESBL-Klebsiella at the admission in all federal states showed a strong drop at the beginning, because we started the simulation with the same prevalence in each hospital, and actually this start prevalence is higher than the final prevalence in many large hospitals (Fig. 4). It is also due to the fact that the decline in many larger hospitals happened faster than the increase in a few small hospitals. After this drop, it increased sharply at the start of each simulation and then gradually reached the final states, which differed across the federal states. It took around 4200 days to reach the final states in Saxony and Thuringia, 6500 days in Bavaria, and 8400 days in Lower Saxony. Lower Saxony had the highest final hospital prevalence (around 1.12–1.27%), closely followed by Bavaria (around 0.94–1.07%), while Saxony and Thuringia had much lower final prevalence than the other two federal states (around 0.54–0.62%). The simulated final prevalences were close to the ESBL-Klebsiella prevalences at admission which were measured in previous studies^20–22^, i.e. $0.85\%$, $0.66\%$, and $1.38\%$. Due to daily admission/discharge patterns, the prevalence in each federal state fluctuated more strongly in hospitals and weaker in community nodes.

**Figure 5:**
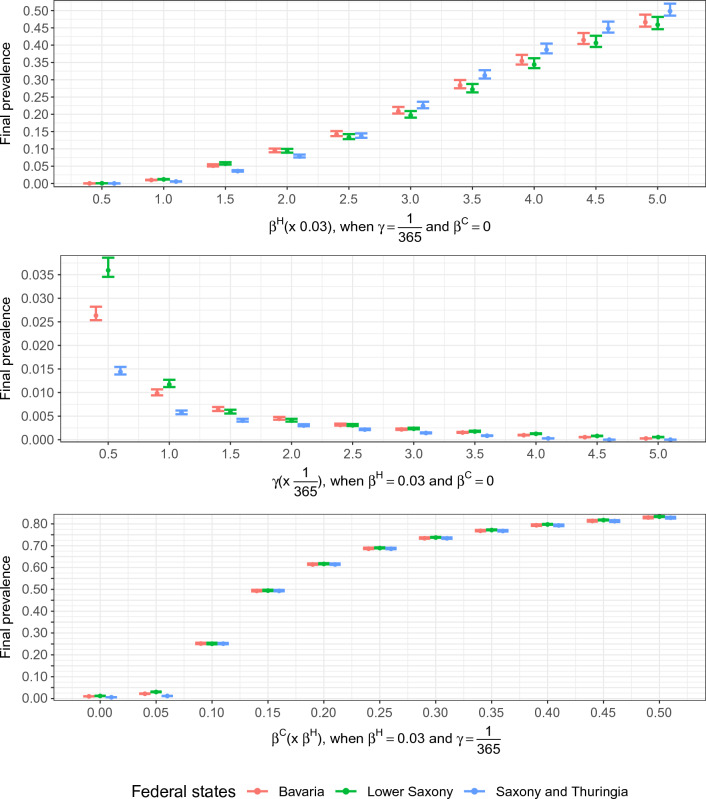
Final average prevalence at admission in hospital nodes for varying transmission parameters in the studied federal states. The upper and lower bars indicate the spread between the highest and lowest prevalence in the weekly patterns. When there is no variation, the bars converge to a single line. $\beta ^H$ and $\beta ^C$ are the transmission rates in hospitals and in community nodes, respectively. $\gamma$ is the colonization clearing rate.

In the next step, we varied the parameters for transmission rate inside hospitals $\beta ^H$, for colonization clearing rate $\gamma$, and for relative community transmission rate $\beta ^C$. We showed in Fig. 5 and Supplemental Fig. S16 that these parameters had different influences on the final prevalence in both hospital and community nodes. In case of $\beta ^C = 0$, the final prevalence in hospital nodes displayed strong variation across days of the weeks. In the case of $\beta ^C > 0.1\cdot \beta ^H$,transmission in the community nodes was decisive for final prevalence, which was shown as the daily fluctuations disappeared and only very little difference between hospital and community prevalence existed. When $\beta ^H$ increased, the final prevalence also increased whereas $\gamma$ had a negative correlation with the final prevalence.

For $\beta ^H\le 2\cdot 0.03$ day$^{-1}$ Lower Saxony had the highest prevalence, whereas the prevalence in Saxony and Thuringia was the lowest. When $\beta ^H$ reached $2.5\cdot 0.03$ day$^{-1}$ Bavaria had the highest prevalence. For a higher $\beta ^H$, the prevalence in Saxony and Thuringia became the highest and the prevalence in Lower Saxony became the lowest. The final prevalence in all federal states was almost 0 in hospital nodes when $\gamma$ reached $5\cdot \frac{1}{365}$ day$^{-1}$. For varying the relative community transmission rate $\beta ^C = \nu \cdot \beta ^H$, the prevalence increased dramatically when $\beta ^C$ ranged from $\beta ^C = 0.05\cdot \beta ^H$ to $\beta ^C = 0.25\cdot \beta ^H$. Afterwards, the increase was slower. Nevertheless, the prevalence was almost the same in the studied federal states after $\beta ^C$ reached $0.1\cdot \beta ^H$.

## Discussion

Based on health insurance data, we constructed hospital networks to study spread of HAI caused by patient transfers between hospitals. The edge weights, defined in “Network construction” of “Methods”, can be regarded as the average epidemic risk in the most severe case caused by the patient transfers in this direction per day. In previous publications^4,6,16^, the edge weights were solely calculated as a sum of the number of patient transfers without differentiating the importance of each transfer and could not efficiently reflect the actual risks in those directions. However, the prevalence in different hospitals might be distinct due to heterogeneous inter- and intra-hospital transfers^23^ as well as measures like hand hygiene etc. We found that differences in network structures were correlated with epidemic outcomes: time to reach the final prevalence and the final prevalence itself in a rather complex way. The prevalence showed a substantially fluctuating pattern. The hospital prevalence was not correlated to the hospital sizes while the LOSs and the hospital prevalence were positively correlated. The final community node prevalence was about 10 times lower than the final hospital prevalence with around $0.09\%$ in Lower Saxony, $0.08\%$ in Bavaria, and $0.05\%$ in Saxony and Thuringia.

Auto-transfers were the dominant risk factor for inter-hospital epidemic spread only when the colonization clearing rate was low. This might be due to the fact that while most of the patient transfers were auto-transfers, the time between hospitalizations in auto-transfers was longer than in other types of patient transfers, thus, after the colonization clearing rate reached a certain value, the other types of transfers contributed more to the pathogen spread.

We first used transmission parameters based on previous research for modeling ESBL-Klebsiella^20–22^ and assumed that the transmission of HAIs does not take place in the community nodes^4,5,11–17^. Therefore, patients who were hospitalized only once played no role in the epidemic spread. This lack of transmission in community nodes is a simplification, but for multi-resistant strains this appears a reasonable approximation. Once also other strains are modeled, transmission in the community can play a stronger role and effectively change the dynamics. We further varied the transmission parameters across a range of clinically relevant parameters for modeling the spread of various HAIs in healthcare systems. From Fig. 5 and Supplemental Fig. S16, the heterogeneous structures of the hospital-community networks in given federal states could be projected into the distinct final prevalence. Ignoring this, because Saxony and Thuringia had the longest average LOS and shortest average number of days between two hospitalizations (see Table 1, Supplemental Fig. S11), we would expect that the prevalence in Saxony and Thuringia would be the highest. However, this was not always true. For varying only the hospital transmission rates $\beta ^H$, when $\beta ^H\le 2\cdot 0.03$ day$^{-1}$, the final prevalence in Lower Saxony was the highest. Bavaria had the highest (among considered federal states) prevalence at $\beta ^H = 2.5\cdot 0.03$ day$^{-1}$. The final prevalence in Saxony and Thuringia took the first place at $\beta ^H\ge 3\cdot 0.03$ day$^{-1}$. With increasing colonization clearing rate $\gamma$, the final prevalence decreased gradually and when $\gamma = 5\cdot \frac{1}{365}$ day$^{-1}$, the ESBL-Klebsiella almost died out. The final prevalence in different federal states was obviously distinct, when the transmission rate inside community nodes was relatively low compared to the transmission rate inside hospitals, i.e. $\beta ^C\le 0.05\cdot \beta ^H$.

The simulated final prevalence based on the initial parameter setup in the simulation was close to the hospital prevalence at admission observed in reality^20–22^. When $\beta ^C$ got larger, the final prevalence in different federal states was close to each other, which indicated that the transmission within community nodes dominated the transmission in the whole system. We found out that even though the transmission rate in community nodes was $10\%$ of the transmission rate in hospital nodes, the hospital prevalence already reached 0.25 which was caused by the fact that the community nodes are much larger than their corresponding hospital nodes.

Our hospital-community based model relied on two assumptions that should be addressed. First, since our data do not include any detailed information about a patient’s residence, we developed community nodes for representing patient stays in real communities. These community nodes are just virtual representations of patients returning at some point to hospitals. We do not have information on people in the community, who do not visit hospitals (within the six years period). As long as we assume no infection transmission in the community, community nodes are just theoretical compartments. Once this assumption is changed, many things change. Real transmissions can only occur among persons with some level of physical proximity and can also occur in overlapping populations, i.e. when from the same population patients attend different hospitals. This can be particularly the case in urban communities served by multiple hospitals. Models accounting for this would require geographical resolution. Furthermore, contacts in community nodes can be diluted by the population not visiting hospitals. Depending on regional age distribution, this segment can vary and the same probability of transmission in the community can result in different prevalence at admission. Furthermore, we were not able to account for regional differences in the population coverage of the studied companies. For example, it could be that the coverage differs between larger cities, smaller cities, and the countryside.

Second, our model assumed homogeneous mixing within the hospitals as well as within community nodes and ignored the real ward structures as well as the community groups interacting with each other. Likely, this heterogeneity results in different transmission dynamics, but addressing this question would require a lot of additional data, not contained in the datasets used in this manuscript. In a different part of the project, we obtained data from single hospitals and conducted intra-hospital modeling^23^, but we were not able to generalize this to all hospitals. In addition, we did not consider other factors potentially influencing transmission besides hospital networks.

Furthermore, we did not account for seasonality. Seasonal variation was described for klebsiella^24^, likely there are also seasonal changes in the hospital transfers and thus in the network structure. Possibly these effects are not strong, but should be assessed in future studies.

In summary, our findings support that in the hospital networks studied here, the spread of multiresistant pathogens caused by patient transfers came mainly from auto-transfers, when the colonization clearing rate was low. The final prevalence differed across federal states due to the mixed effects of heterogeneous patient characteristics and the structures of constructed hospital-community networks. Assuming pathogen parameters of ESBL-Klebsiella, Saxony and Thuringia would have the lowest prevalence. However, for other HAIs with a higher hospital transmission rate, Saxony and Thuringia would have higher prevalence than the other two federal states. Thus, transmission parameters of the pathogen might determine which network organization performs better in reducing epidemic risk.

## Methods

### Creating federal scaled-up datasets

Due to the multitude of insurance companies in Germany^25^, we had to rely on datasets that do not cover the entire population. We used six-year anonymized hospital discharge databases, provided by three German regional insurance companies: AOK Bavaria (active in the federal state of Bavaria), AOK Lower Saxony (in Lower Saxony), and AOK PLUS (in Saxony and Thuringia). Since the dataset from AOK PLUS included patients from two federal states that could not be separated because of unknown hospital locations, three regions were compared, even if four federal states were included. AOKs (Allgemeine Ortskrankenkasse, historically “general local healthcare insurance company”, but currently only the abbreviation is used) are insurance companies, which historically exclusively insured persons from the federal states where they were founded. Therefore, they have high coverage of the population in their own federal state and low coverage outside. The data cleaning process, which ensures the unique assignment of patients to hospitals every single day, is described in our previous research^4^.

AOK-insured persons were divided into sex and age groups and compared to the general population in the corresponding federal state to obtain age- and sex-specific population coverage (see Supplemental Figs. S1–S3). According to our previous research^4^, the distribution of the length of hospital stay (LOS) varied between sex and age groups. The crude analysis of the AOK patient records can lead to an inaccurate estimation of LOS and patient transfer patterns, which would affect the simulation results of epidemic spread. Nevertheless, the population coverage of our AOK datasets is generally above $30\%$ in each sex and age group, and the general patient transfer patterns can still be recovered as shown in^4^. To reduce the effect of a non-uniform sex and age distribution in the populations of the insurance companies, we reweighted their populations to fit the sex and age distribution of the respective federal states^4^. In brief, for each sex and age group, we randomly resampled patients from the same sex and age group in the AOK datasets to compensate for patients that were not insured by AOK. For each resampling, once the patient was chosen, his/her hospitalization records were duplicated and labeled as a new patient. The resampling procedure was repeated within each sex and age group until there was the same number of patients in the resampled dataset as there was in the corresponding total population of the federal state. We labeled these datasets as federal scaled-up datasets. We generated 100 different federal scaled-up datasets for each federal state and used them for all further analyses in corresponding federal states. More detailed information about the original AOK datasets can be also assessed in our previous analyses^4,5,26,27^.

### Network construction

We used federal scaled-up datasets to build two networks to represent the patient transfer data. The first “hospital network” was not used for detail modeling epidemic spread but only for reflecting the importance of each patient transfer path within a certain period. This network had *N* nodes and *e* directed weighted edges, where nodes represented the hospitals. An edge connecting node *i* to node *j* indicated the existence of patient transfers from the *i*-th to the *j*-th hospital, and its weight was defined as $m_{ij} = \epsilon = \frac{\sum _{\epsilon = 1}^{L_{ij}}e^{-\gamma \Delta t_{\epsilon }}}{T}$. Here, $\epsilon \in \big \{1,\dots ,L_{ij}\big \}$ denoted each patient transfer from *i*-th to *j*-th node, and $\Delta t_{\epsilon }$ was the duration of transfer $\epsilon$ between discharge from hospital *i* and admission to hospital *j*. The term $e^{-\gamma \Delta t_{\epsilon }}$ indicated the probability of a patient colonized with a HAI at discharge still carrying HAI at the next admission^13,15^. The parameter $\gamma$ was the colonization clearing rate and *T* was the observation period determined by the data. $L_{ij}$ indicated the total number of transfers from *i*-th to *j*-th node during the time interval [1, *T*].

Initially, to model the spread of Extended-spectrum $\beta$ lactamase-Producing Klebsiella pneumoniae (ESBL-Klebsiella)^20–22^ and other common HAIs^4,5,11–17,28^, we set $\gamma = \frac{1}{365}{\,\text {day}}^{-1}$ based on the assumption that the meantime of colonization was 365 days in the baseline scenario. We also studied other patient transfer patterns for different values of $\gamma$ with $\gamma = \vartheta \cdot \frac{0.5}{365}\,\text {day}^{-1}$, $\vartheta = 0,1,2,\dots ,300$ for sensitivity analyses. Note that for $\gamma = 0$, the edge weight represents the average number of transfers per day between considered hospitals; we defined this as total edge weights. The effective edge weight $m_{ij}$ indicated the average number of colonized patients per day, which can transmit the disease from node *i* to node *j*. The effective edge weights can be viewed as a measure of average daily transmission risk from node *i* to node *j*. The total edge weights and effective edge weights differ for $\gamma \ne 0$, because some of the transfers may not lead to a transmission when the patient has cleared colonization before being readmitted. Weight $m_{ii}$ described the situation of patients being readmitted to the same hospitals, and we defined these kinds of patient transfers as auto-transfers.

**Figure 6:**
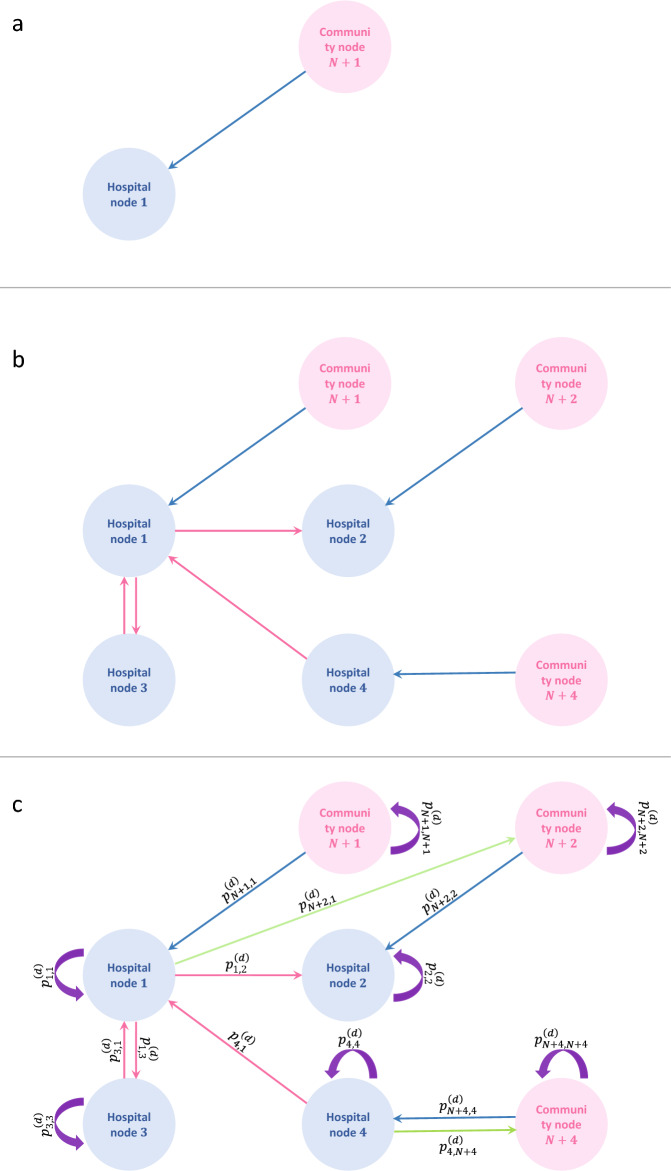
Representation of hospital-community network on a day *d* concerning possible patient movements between two hospitals and corresponding communities. Panel (**a**) shows the admission of patients to the hospital from their corresponding community. Panel (**b**) shows that patient transfers can also happen between hospitals. Panel c shows that patients can be transferred from hospitals to communities. Links between nodes indicate all possible routes of patient transfers. Purple loops indicate the situations when patients stay in the hospital or community nodes.

The second network was generated to simulate the patients’ residence outside hospitals, which we used for modeling the daily patient transfers and epidemic spread of the studied pathogen. We refer to it as a “hospital-community network” (Fig. 6).

Since the anonymized AOK data did not contain any information about hospital locations and in order to track the patient staying at home between successive hospitalizations, for each hospital *i* we created a community-node indexed by $N+i$, where *N* was the number of all considered hospitals^4,28^. However, in contrast to the previous approach^28^, we assumed the patients to be in the community-node $N+i$ if their next hospitalization was at hospital *i*. After the last discharge, the patient was allocated to the corresponding community-node of the hospital, where the last hospitalization had occurred. Since hospital admission and discharge patterns were heterogeneous on different days of the week (see Supplemental Fig. S4), we created separate hospital-community networks for each day of the week $w\in \{1,\dots ,7\}$, representing Monday to Sunday. In this network, edges represented all possible routes of patient transfers between the nodes (hospital- or community-nodes). They were weighted by ${p_{ij}}^{(w)} = \frac{n_{ij}(w)}{a_i(w)}$ with $i \ne j$, describing the probability of a patient being transferred from node *i* to *j* on a day of the week *w*, where $n_{ij}(w)$ with $i\ne j$ denoted the average number of transferred patients from node *i* to *j* on days of the week *w*, and $a_i(w)$ represented the average number of patients in node *i* on one day of the week *w*, both calculated directly from federal scaled-up datasets. Similarly to the previous approach^28^, the probability of a patient staying in node *i* on one day of the week *w* can be calculated as $p_{ii}^{(w)} = 1 - \sum _{j = 1,j\ne i}^{2N}p_{ij}^{(w)}$. The hospital-community network on one day of the week *w* can also be simply presented by a transfer probability matrix ${{\textbf {P}}}^{(w)} := \left[ p_{ij}^{(w)}\right] _{i,j = 1}^{2N}$, with $w = 1,\dots ,7$.

### Sizes of hospital nodes

Since healthcare facilities were anonymized, we did not have external information on their sizes. Thus, to estimate the sizes of considered hospital nodes, we counted the number of occupied beds in the federal scaled-up data in each facility each day, and then took the average over the facility’s opening period. Analogously, we also defined the sizes of corresponding community-nodes. To deliver a better overview of hospital properties, we can summarize hospital properties by using three size groups: large (*L*), medium (*M*) and small (*S*) (see Table 2), based on definition used in previous research^29^.

**Table 2:** Distribution of hospitals based on size.

Hospital size		Large (*L*)	Medium (*M*)	Small (*S*)
Stratification criterion*		$\ge 800$	[400, 799]	$\le 399$
Number of hospitals	Bavaria	19	34	304
	Lower Saxony	5	31	175
	Saxony and Thuringia	8	16	102

$^{*}$ Stratification criterion: average number of occupied beds.

### Analysis of hospital networks

In the hospital networks, two types of transfers were observed: the first type—called auto-transfers—described the event that patients were readmitted to the same hospitals^5,28^; the second type described transfers between different hospitals.

For the hospital networks, effective edge weights reflected the transmission risk through patient transfers between hospitals. Thus, the risk of acquiring and spreading the infection in a hospital can be measured by summing the effective weights of its ingoing and outgoing edges, defined as in- and out-strengths, respectively. Since hospital size determined the capacity for patient admissions and patient transfers, we calculated the strengths of single hospitals and their connected hospital size groups, defined in Table 2. The in-strengths of hospital *i* can be caused by auto-transfers and transfers arising from hospitals in hospital group *G*, defined as $s_{i\rightarrow i} = m_{ii}$ and $s_{G\rightarrow i} = \sum _{j\in G\wedge j\ne i}m_{ji}$, respectively. Similarly, the out-strength of hospital *i* can also be caused by auto-transfers $s_{i\rightarrow i}$ and transfers targeting to hospitals in hospital group *G*, defined as $s_{i \rightarrow G} = \sum _{j\in G\wedge j\ne i}m_{ij}$ with $G = L, M, S$ as defined above. To investigate the overall distribution of strengths, we also calculated the total in- and out-strengths by summing the strengths of every hospital by distinguishing different transfer types.

### Modeling HAI spread based on hospital-community networks

We used the hospital-community networks described in “Network construction” to simulate the patient traffic and spread of the pathogen in the networks.

The main features of the proposed model were:

- Nodes in the network represented healthcare facilities and communities.
- The pathogen spread in the nodes was modeled using the standard SIS model.
- In the nodes, admissions, discharges and transfers occurred. They are assumed to happen instantly at the end of every day.
- Admissions, discharges, and transfers were the subject to day-to-day fluctuations depending on day of the week, which was accounted for in the model.

The rest of this section refers to the mathematical description of the model displaying these features. Let $c^0 := \left[ {c_1}^0,\dots ,{c_{2N}}^0\right]$ be the initial patient probability distribution in hospital-nodes (indexed from 1 to *N*) and community-nodes (indexed from $N+1$ to 2*N*) at the end of day 1 (Monday) i.e. a vector with the coordinates from 1 to *N* storing the probability of a patient to be in a particular hospital (numbered from 1 to *N*), while the coordinates from $N+1$ to 2*N* storing the probability of a patient to be in a particular community (numbered from $N+1$ to 2*N*). The model operates on the probability distributions, i.e., on the ratios of the number of patients in each particular node compared to the total number of patients present in the system. Therefore instead of the absolute patient numbers, the sum of all elements of vector $c^0$ is 1. Multiplying the patient probability distribution vector by the total number of patients in the system we can restore the numbers of patients in particular hospital or community nodes. The patient probability distribution at the end of day *d*, denoted as $c^d$, is calculated by multiplying the patient probability distribution vector at the day before (i.e. $c^{d-1}$) by the transfer probability matrix at day *d* (i.e. ${\textbf {P}}^d$):

$$\begin{aligned} c^d:= c^{d-1}{} {\textbf {P}}^d. \end{aligned}$$

We assumed that $d=1$ represents Monday, $d=2$ represents Tuesday and so on while $d=7$ represents Sunday. We can repeat that calculation *d* times obtaining

$$\begin{aligned} c^d=c^0{\textbf {P}}^1\cdot \ldots \cdot {\textbf {P}}^d. \end{aligned}$$

Thus, function *m*, defines as $m(d)=\frac{\left( d-(d\mod 7)\right) }{7}$, gives us the number of full weeks within the *d* days of simulation. So the patient probability distribution at day *d* is given by

$$\begin{aligned} c^d:= c^0{\textbf {P}}^1\cdot \ldots \cdot {\textbf {P}}^d = c^0\left( {\textbf {P}}^1\cdot \ldots \cdot {\textbf {P}}^7\right) ^{m(d)}\cdot {\textbf {P}}^1\cdot \ldots \cdot {\textbf {P}}^{(d\mod 7)} = c^0({\textbf {P}})^{m(d)}\cdot {\textbf {P}}^1\cdot \ldots \cdot {\textbf {P}}^{(d\mod 7)}. \end{aligned}$$

Clearly, if $(d\mod 7) = 0$, this means that we simulate multiple weeks, then the formula can be written as

$$\begin{aligned} c^d:= c^0\left( {\textbf {P}}^1\cdot \ldots \cdot {\textbf {P}}^7\right) ^{m(d)}:= c^0({\textbf {P}})^{m(d)}, \end{aligned}$$

where weekly transfer matrix $${\textbf {P}}$$ is defined as $${\textbf {P}}:= 
{\textbf {P}}^1\cdot \ldots \cdot {\textbf {P}}^7$$.

To define the initial condition for our simulations, first we found the week-stationary patient probability distribution which indicates the expected long-term distribution of patient probabilities. To do so, we used weekly transfer matrix ${\textbf {P}}$ and confirmed that it was regular. Next, we found the left eigenvector $c^{\star }$ of matrix ${\textbf {P}}$ corresponding to eigenvalue 1 by solving system $c{\textbf {P}} = c$. Finally, we set $c^0 = c^{\star }$ as the initial condition for our model.

We used ESBL-Klebsiella as an exemplary multiresistant pathogen and modeled the colonization of patients within nodes. We followed^5,28^ and adopted a susceptible–infectious–susceptible (SIS) ordinary differential equations model. We assumed that each node has one well-mixed and constant in time population consisting of *N* individuals that can be either susceptible (*S*) or infectious (*I*), thus $I(d) + S(d) = N$ where *d* denotes time (counted in days). Here, patients colonized or infected were defined as “infectious”. Since the group of colonized patients is much larger than the group infected, we use the term colonized and infectious interchangeably. The transmission of the pathogen takes place as a result of the contact of susceptible and infectious individuals and is described by the term $\beta \frac{I(d)}{N}S(d)$, while the process of clearing colonization is modeled by the term $\gamma I(d)$. Thus, the system describing the daily change of the number of patients in each stage (susceptible and infectious) is given by:

1 $$\begin{aligned} \frac{\partial }{\partial d}I(d)&=\beta \frac{I(d)}{N}S(d)-\gamma I(d),\end{aligned}$$

2 $$\begin{aligned} \frac{\partial }{\partial d}S(d)&=-\beta \frac{I(d)}{N}S(d)+\gamma I(d). \end{aligned}$$

Since $I(d) + S(d) = N$, we have

3 $$\begin{aligned} \frac{\partial }{\partial d}I(d) = -\beta \frac{\left( I(d)\right) ^2}{N} + (\beta - \gamma )I(d), \end{aligned}$$

which is the logistic equation^30^ with analytical solutions, which exist globally and are unique. We applied this SIS model to all hospital- and community-nodes. However, since we focus only on Klebsiella as an example of HAIs in our work, we assumed that the transmission rate in all community nodes $\beta ^C$ was equal to zero in the baseline scenario. In contrast, the transmission rate in hospital nodes $\beta ^H$ was set to $\beta ^H = \beta > 0$.

All individuals (at the end of day *d*, for $d\in N^{+}$) were assigned to one of two groups (susceptible or infectious), thus vectors $I^d:= \left[ {I_1}^d,\ldots ,{I_{2N}}^d\right]$, $S^d := \left[ {S_1}^d,\ldots ,{S_{2N}}^d\right]$ contain the number of patients of each group in each hospital and each community. The total number of patients at day *d* in each hospital and community as given by a vector

$$\begin{aligned} N^d:= \left( S^0+I^0\right) \cdot ({\textbf {P}}^1\cdot \ldots \cdot {\textbf {P}}^7)^{m(d)}\cdot {\textbf {P}}^1\cdot \ldots \cdot {\textbf {P}}^{(d\mod 7)} = S^d+I^d, \end{aligned}$$

which can be rewritten as

$$\begin{aligned} N^d:= \left( S^0+I^0\right) \cdot ({\textbf {P}})^{m(d)}\cdot {\textbf {P}}^1\cdot \ldots \cdot {\textbf {P}}^{(d\mod 7)}. \end{aligned}$$

Let *n* denote the total number of patients on the first day in the system. Then, since $I^0 + S^0 = n\cdot c^0$, we have $N^d := n\cdot c^0\cdot ({\textbf {P}})^{m(d)}\cdot {\textbf {P}}^1\cdot \ldots \cdot {\textbf {P}}^{(d\mod 7)}$. If we start with the eigenvector $c^0=c^{\star }=c^{\star }{} {\textbf {P}}$, then $N^d := n\cdot c^{\star }\cdot ({\textbf {P}})^{m(d)}\cdot {\textbf {P}}^1\cdot \ldots \cdot {\textbf {P}}^{(d\mod 7)}$.

Our algorithm was implemented in R and it is as follows: first, we set initial conditions $I^0 := \left[ {I_1}^0,\dots ,{I_{2N}}^0\right]$, $S^0 := \left[ {S_1}^0,\dots ,{S_{2N}}^0\right]$ such that the number of individuals in nodes agreed with the distribution given by week-stationary patient i.e. $I^0 + S^0 = n\cdot \left[ {c_1}^0,\dots ,{c_{2N}}^0\right]$. Then for each node we solved the logistic equation (3) for 1 day duration numerically (indicated as solveLogistic function), calculated numbers of susceptible patients and then transferred the obtained numbers of patients in each group in each node according to a given day matrix. We repeated that procedure for *D* days, as presented below:
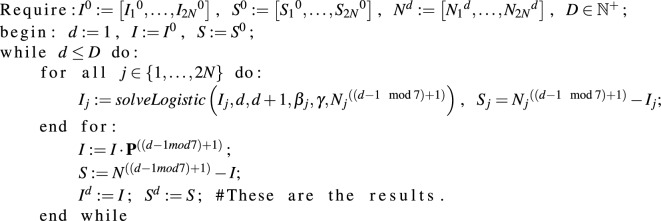


We set the simulation time-step as one day, and the colonization clearing rate and transmission rate did not vary between hospitals. For the initial conditions in our simulation, we adopted the week-stationary probability distribution of patients within the nodes and assigned the start prevalence as 0.005 in all hospital nodes and 0.0005 in all community nodes. We chose $D=14{,}600$ days for the simulation length. Here, if the prevalence gets into a stable periodicity, we say that the prevalence reaches the final state.

Parameters for modeling ESBL-Klebsiella were based on the literature^20–22^, i.e. $\beta ^H = 0.03$ day$^{-1}$ for transmission rate within hospitals and $\beta ^C = 0$ for transmission inside community nodes, while the colonization clearing rate was identical in hospital and community nodes, set as $\gamma = \frac{1}{365}$ day$^{-1}$. Apart from these default parameter settings, we varied the parameter values to study the behavior of our model. The $\beta ^H$ and $\gamma$ were varied by setting $\beta ^H = p\cdot 0.03$ day$^{-1}$ and $\gamma = q\cdot \frac{1}{365}$ day$^{-1}$ with $p,q = 0.5, 1,\dots , 5$.

In the literature it has been pointed out that several HAIs, for instance Methicillin-resistant Staphylococcus aureus, can also be transmitted within communities^31^. With this concern, in addition to the original parameter settings, we varied the parameter for community transmission rate $\beta ^C$ by setting $\beta ^C = \nu \cdot \beta ^H$ with $\nu = 0, 0.05,\dots , 0.5$. Since we here assumed that $\beta ^C$ is proportional to $\beta ^H$, we named it “relative community transmission rate”. We have generated 100 different federal scaled-up datasets and obtained a probability matrix for each dataset. Then we conducted simulations by busing the average of these probability matrices.

### Supplementary Information

Supplementary Information.

## Data Availability

The anonymized insurance data are owned by a third party (AOK Lower Saxony) and authors do not have permission to share them. These data may be requested from: AOK Bavaria, Carl-Wery- Straße 28, 81739 München, https://www.aok.de/pk/bayern/; AOK Lower Saxony, Hildesheimer Straße 273, 30519 Hannover, https://niedersachsen.aok.de/; AOK PLUS, Sternplatz 7, 01067 Dresden, https://www.aok.de/pk/plus/. For further questions, please contact the corresponding author.

## Code availability

The code for the data processing, data analyzing, and SIS simulations was written in R. To visualize the data we used the R package “ggplot2”^32^. For all analyses, we used R version 4.0.3^33^. The original R codes are uploaded to GitHub under the URL: https://github.com/SchroedingerKatze/EmergeNetComparison.
