# Supplementary material for: Regional patient transfer patterns matter for the spread of hospital-acquired pathogens
Source: Sci Rep. 2024 Jan 9;14:929. doi: 10.1038/s41598-023-50873-z (PMC10776674; doi:10.1038/s41598-023-50873-z)
Supplement: Supplementary file 1 — Supplementary Information. [file 41598_2023_50873_MOESM1_ESM.pdf]

Regional patient transfer patterns matter for the spread of hospital-acquired pathogens

Hanjue Xia[1,*], Johannes Horn[1], Monika J. Piotrowska[2], Konrad Sakowski[2], André Karch[3], Mirjam Kretzschmar[4], Rafael Mikolajczyk[1]

1 Institute for Medical Epidemiology, Biometrics and Informatics (IMEBI), Interdisciplinary Centre for Health Sciences, Medical School of the Martin Luther University Halle-Wittenberg, Halle, Saxony-Anhalt, Germany

2 Institute of Applied Mathematics and Mechanics, University of Warsaw, Poland

3 Institute for Epidemiology and Social Medicine, University of Münster, Münster, North Rhine-Westphalia, Germany

4 Julius Centre for Health Sciences & Primary Care, University Medical Centre Utrecht, Utrecht University, Utrecht, The Netherlands

*hanjue.xia@uk-halle.de

Supplementary Results

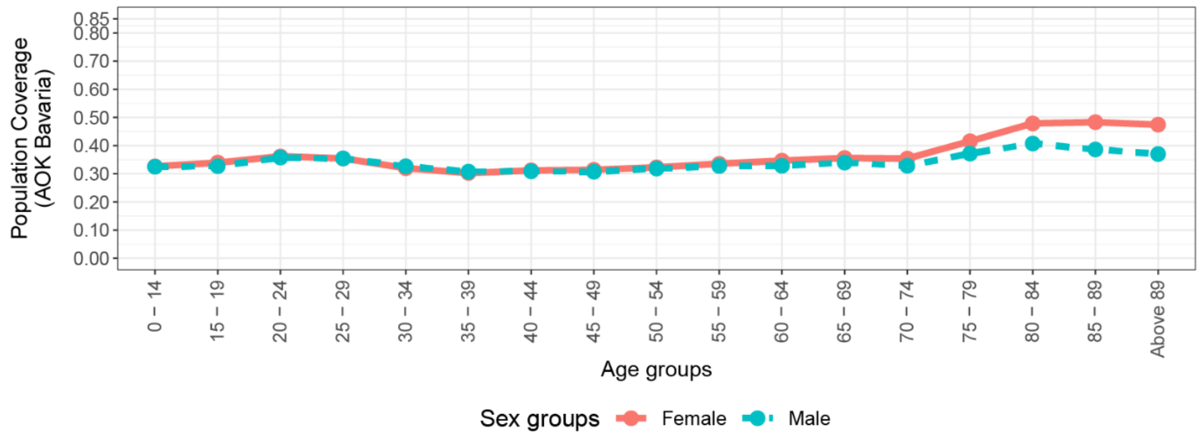

Figure S1. Proportions of the population covered by AOK according to different sex and age groups in Bavaria.

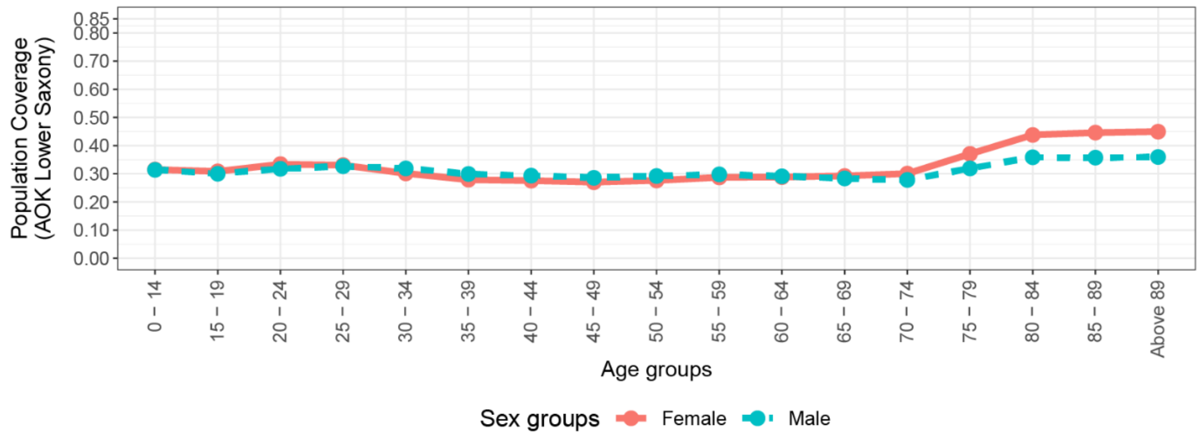

Figure S2. Proportions of the population covered by AOK according to different sex and age groups in Lower Saxony.

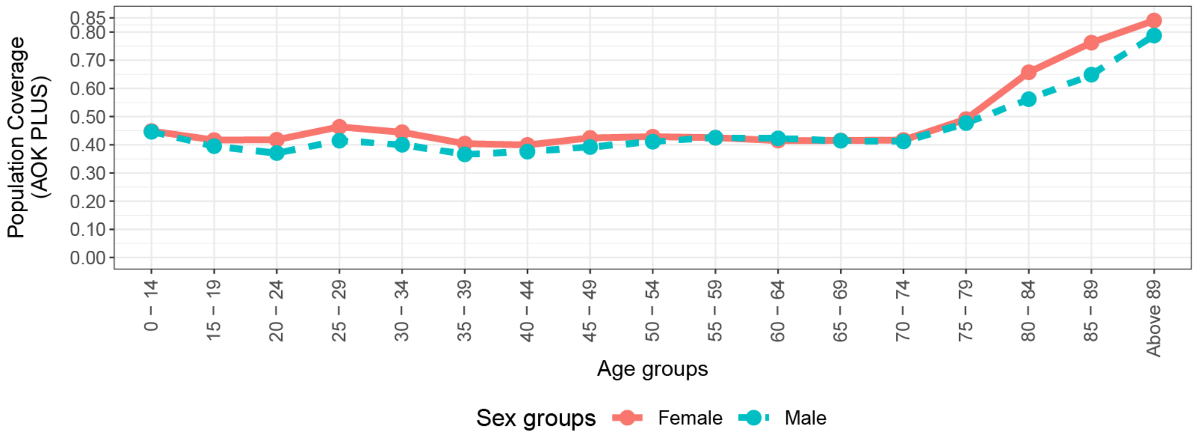

Figure S3. Proportions of the population covered by AOK according to different sex and age groups in Saxony and Thuringia.

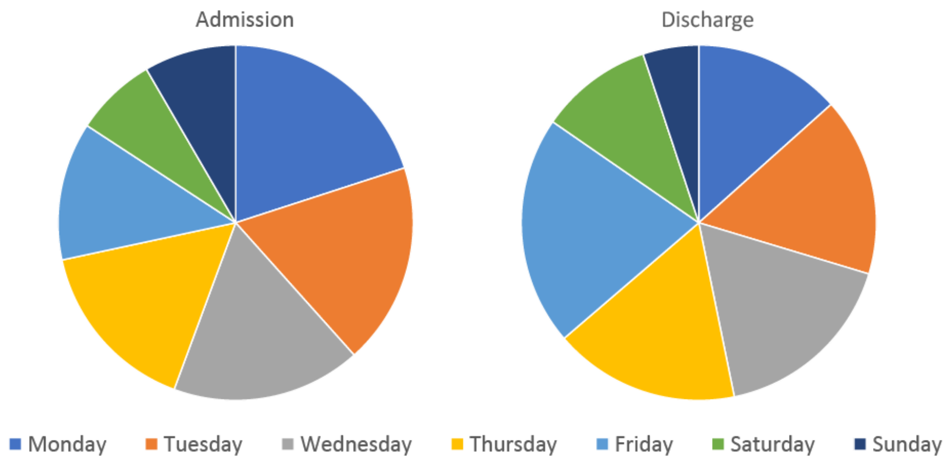

Figure S4. Hospital admissions and discharges on different days of the week based on federal scaled-up datasets.

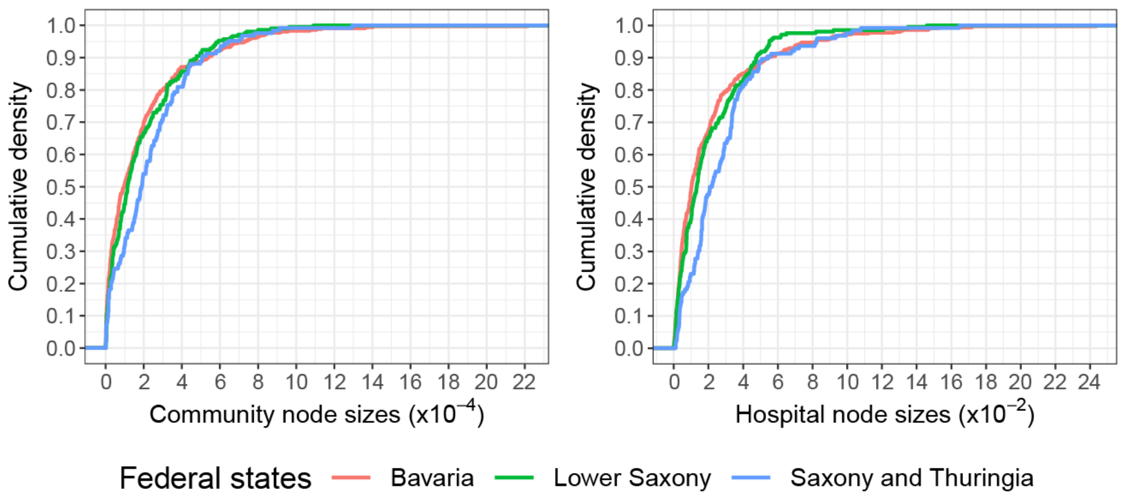

Figure S5. Cumulative distributions of hospital and community node sizes in different federal states based on federal scaled-up datasets.

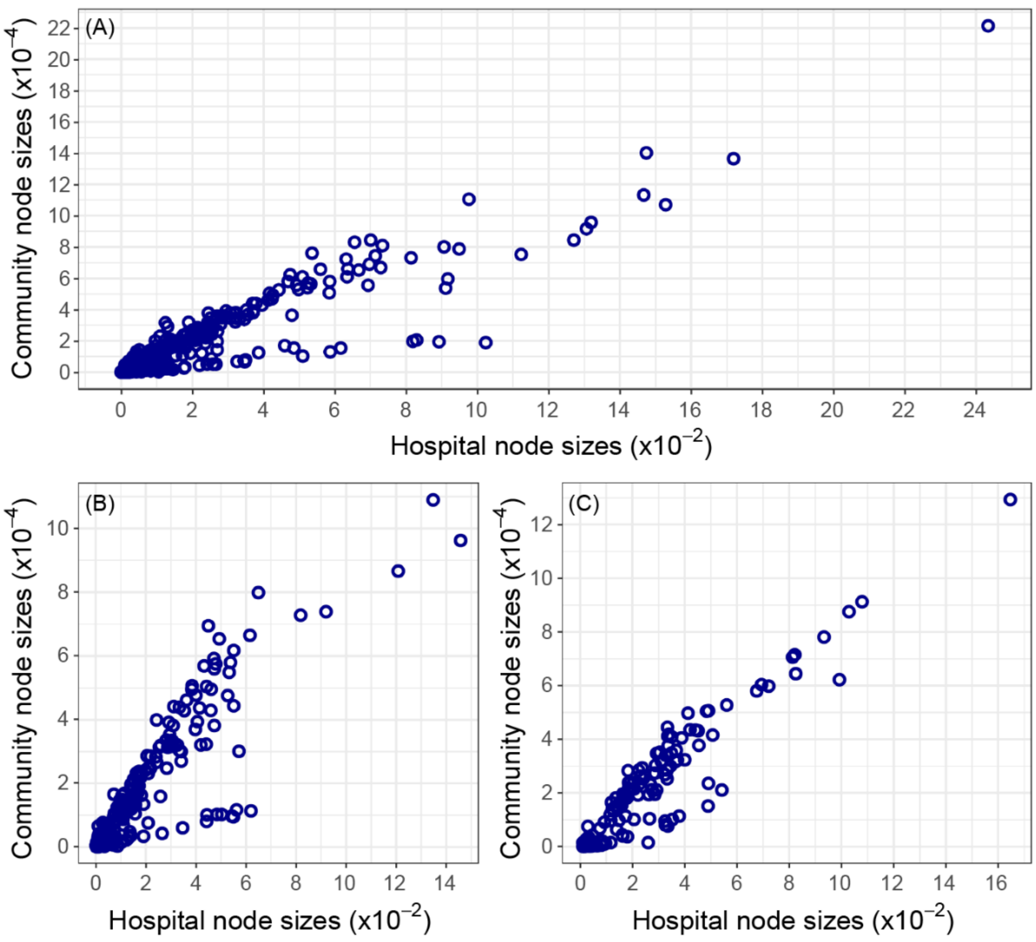

Figure S6. Dependencies of corresponding community node sizes on hospital node sizes in Bavaria (panel a), Lower Saxony (panel b), Saxony and Thuringia (panel c) based on federal scaled-up datasets.

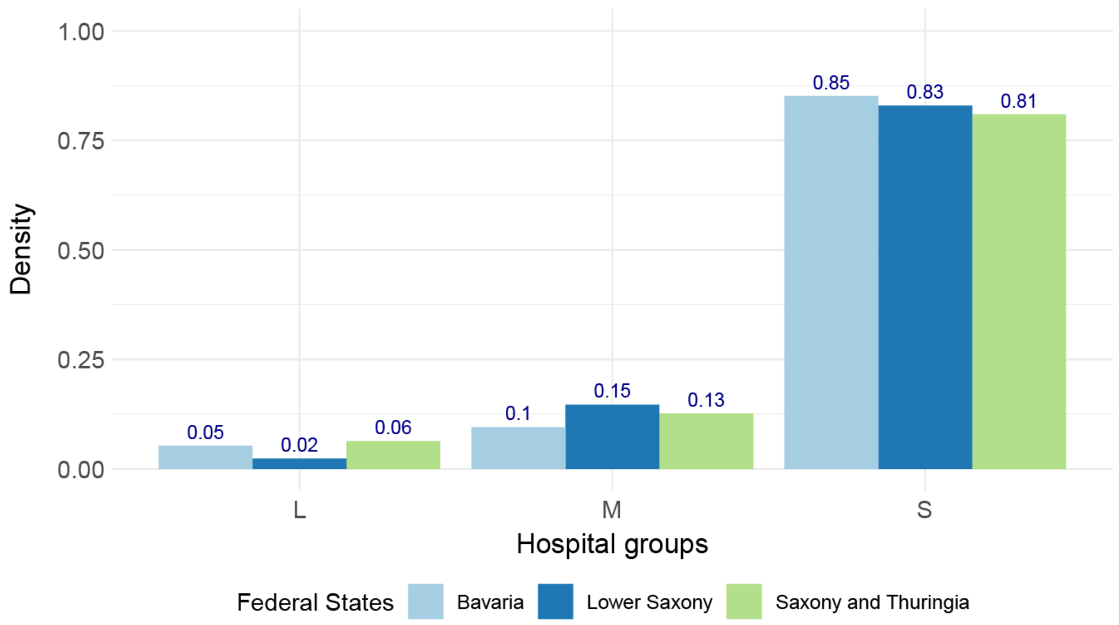

Figure S7. Proportions of hospitals according to hospital sizes L (Large), M (Medium), S (Small) by federal states based on federal scaled-up datasets.

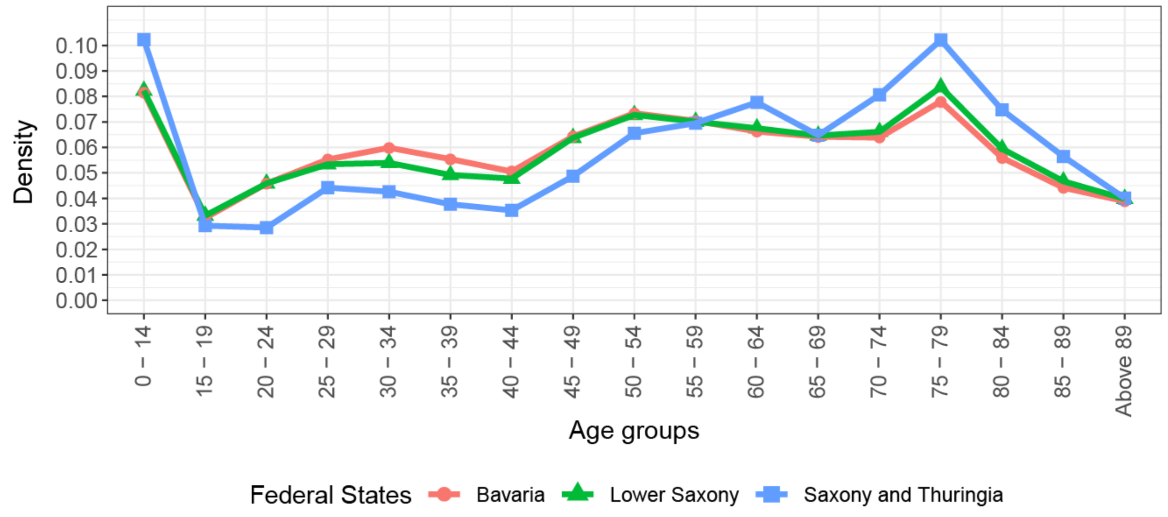

Figure S8. Distributions of admissions in the age groups by federal states based on federal scaled-up datasets.

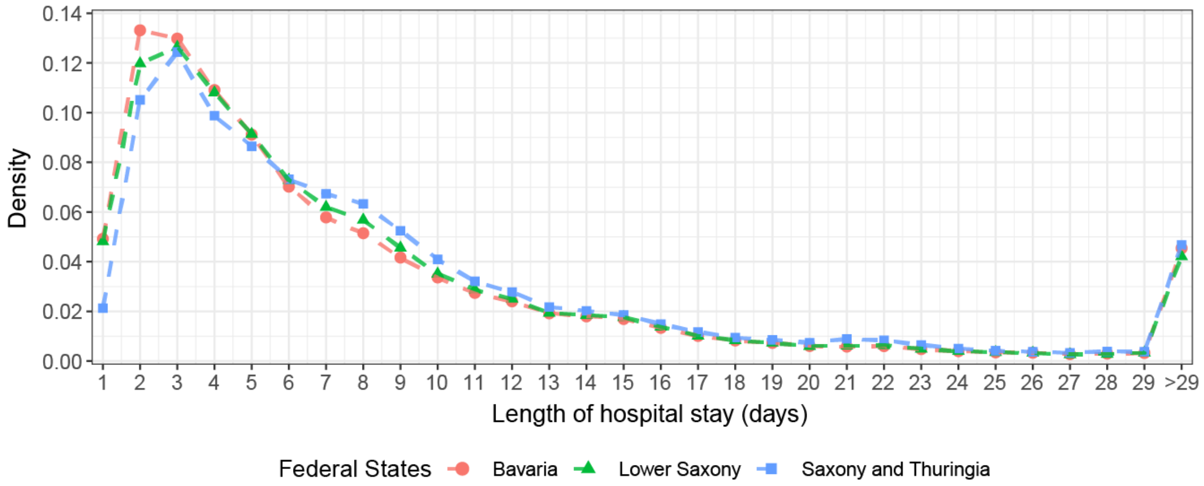

Figure S9. Distributions of the length of hospital stays by federal states based on federal scaled-up datasets.

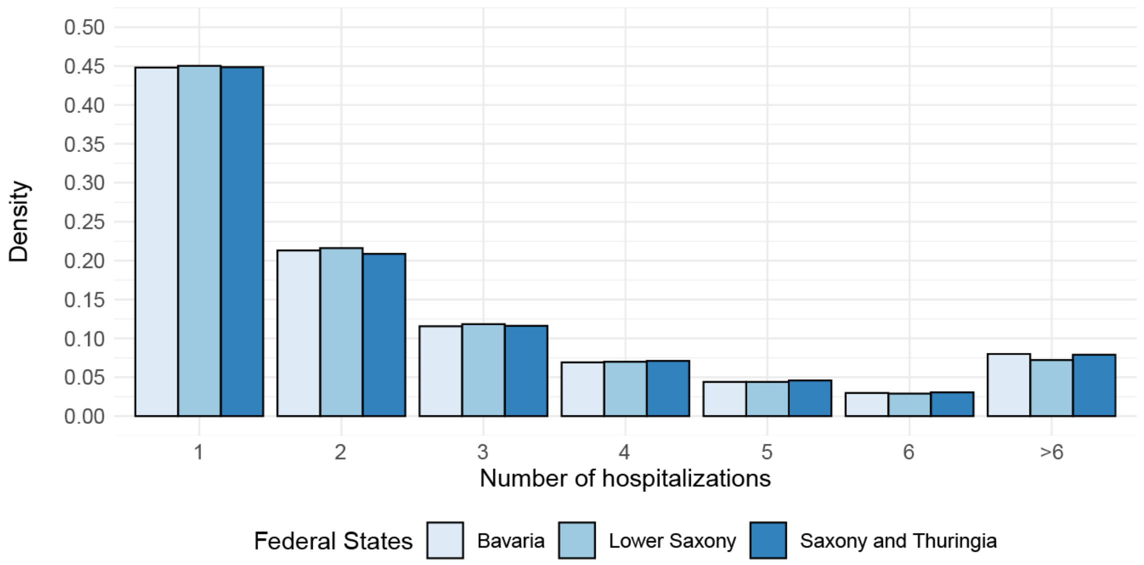

Figure S10. Distributions of the number of hospitalizations per person within six years by federal states based on federal scaled-up datasets.

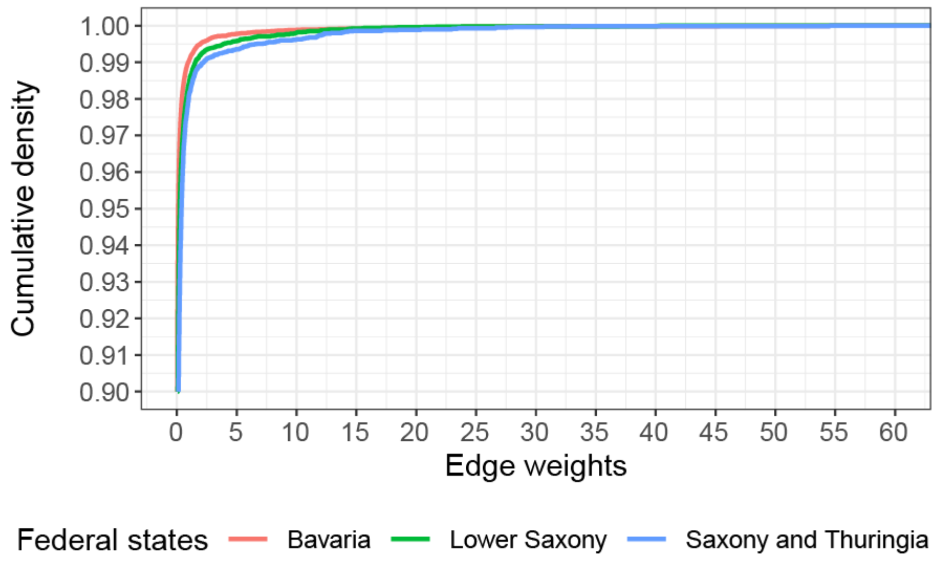

Figure S11. Cumulative distributions of edge weights in hospital networks by federal states based on federal scaled-up datasets.

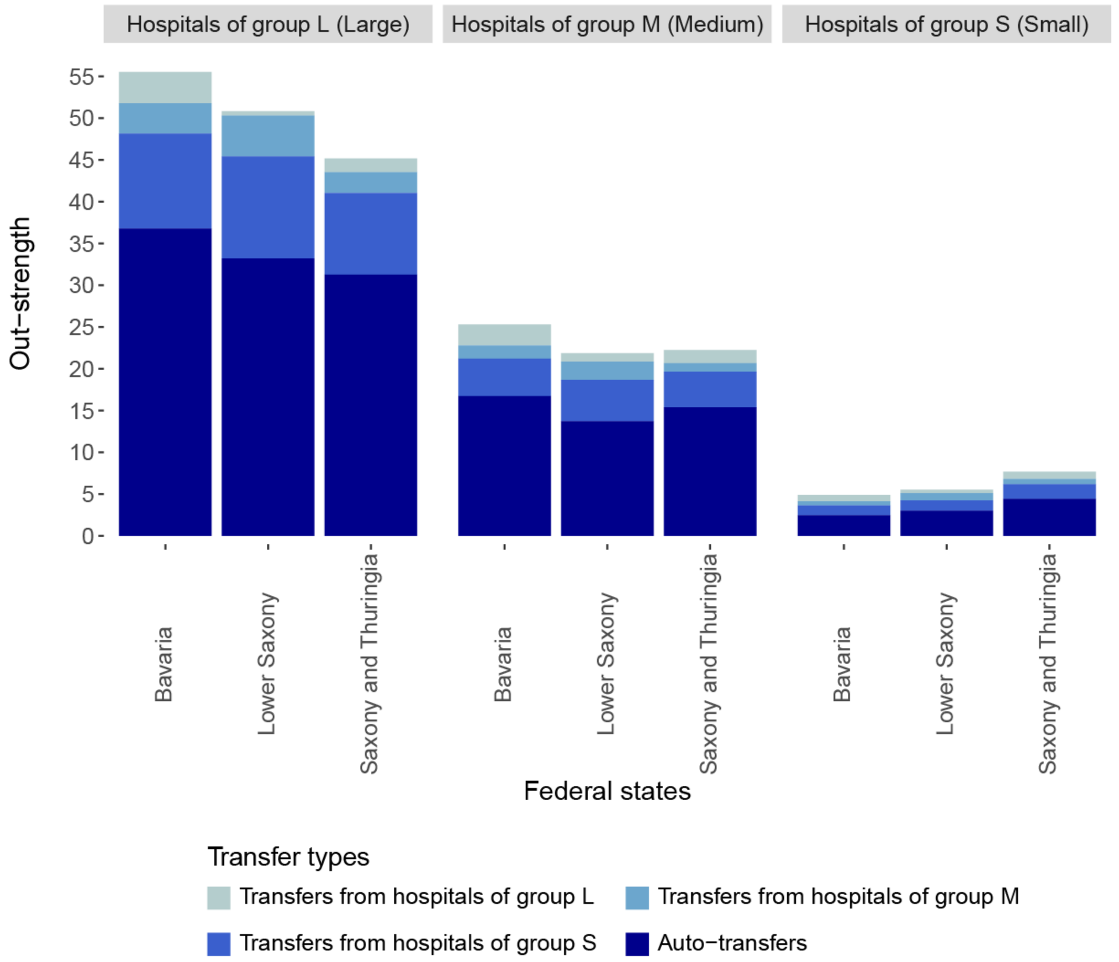

Figure S12. Out-strengths for hospitals originated from auto-transfers and from transfers from different hospitals, categorized by hospital sizes L (Large), M (Medium), S (Small). The x-axis represents the corresponding federal states.

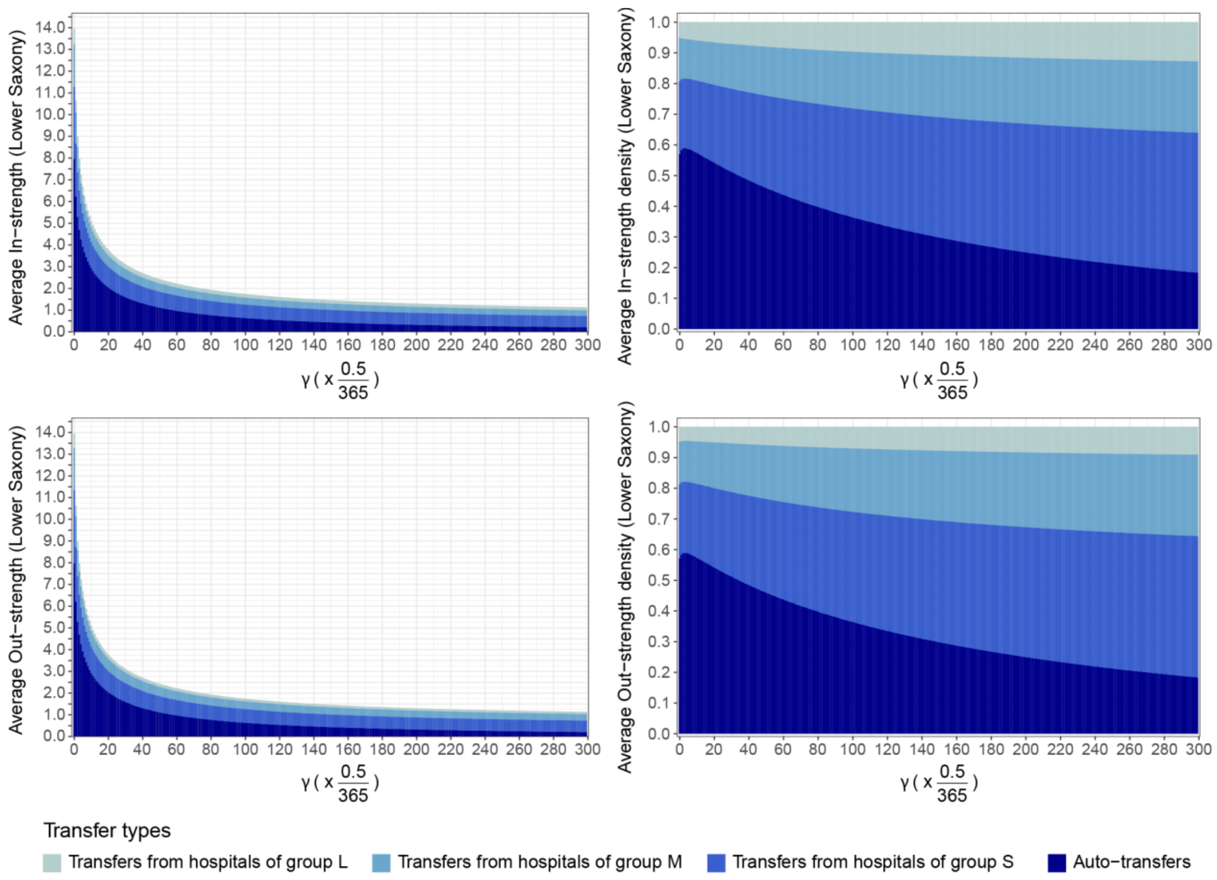

Figure S13. Distributions and density function of average in- and out-strengths of hospitals originated from auto-transfers and from hospitals categorized by hospital sizes (L, M, S) with varying colonization clearing rate γ in Lower Saxony.

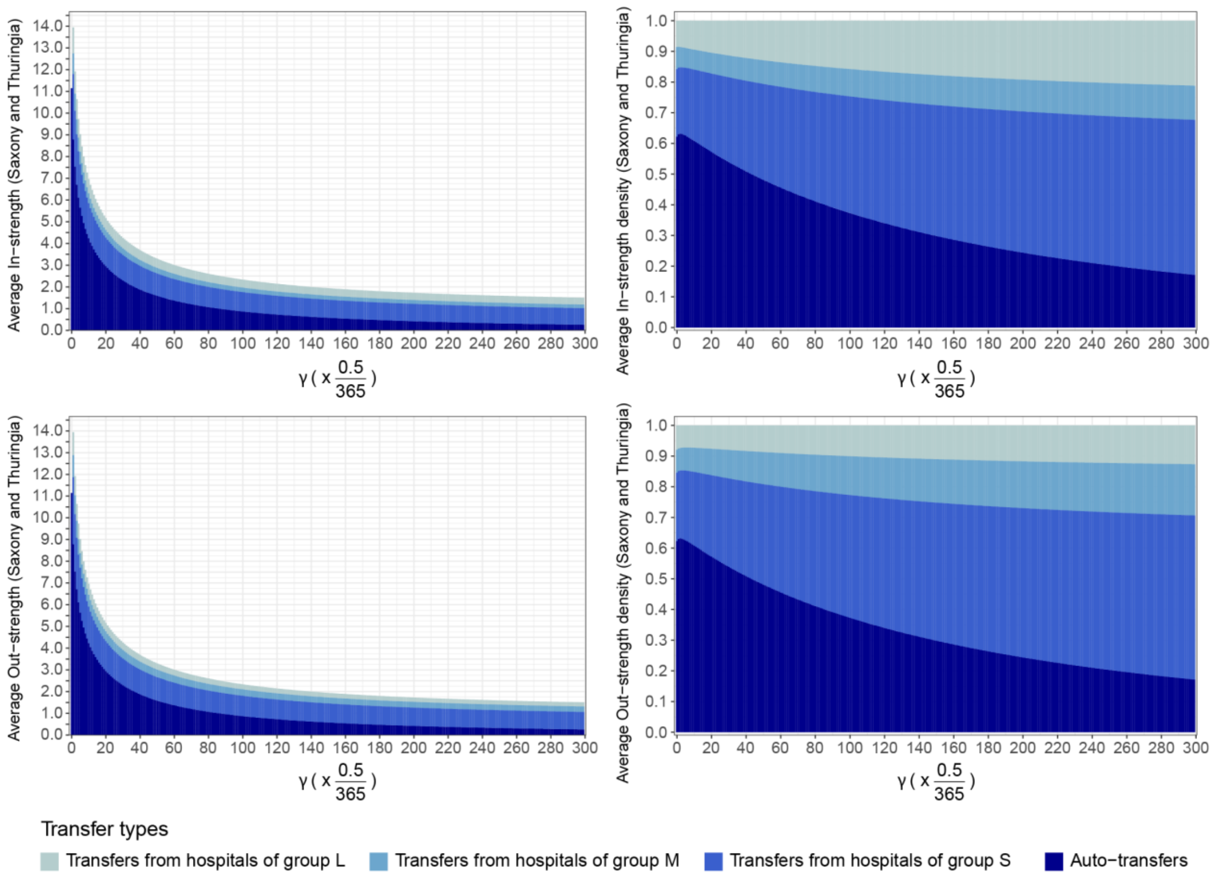

Figure S14. Distributions and density functions of average in- and out-strengths of hospitals originated from auto-transfers and from hospitals categorized by hospital sizes (L, M, S) with varying colonization clearing rate γ in Saxony and Thuringia.

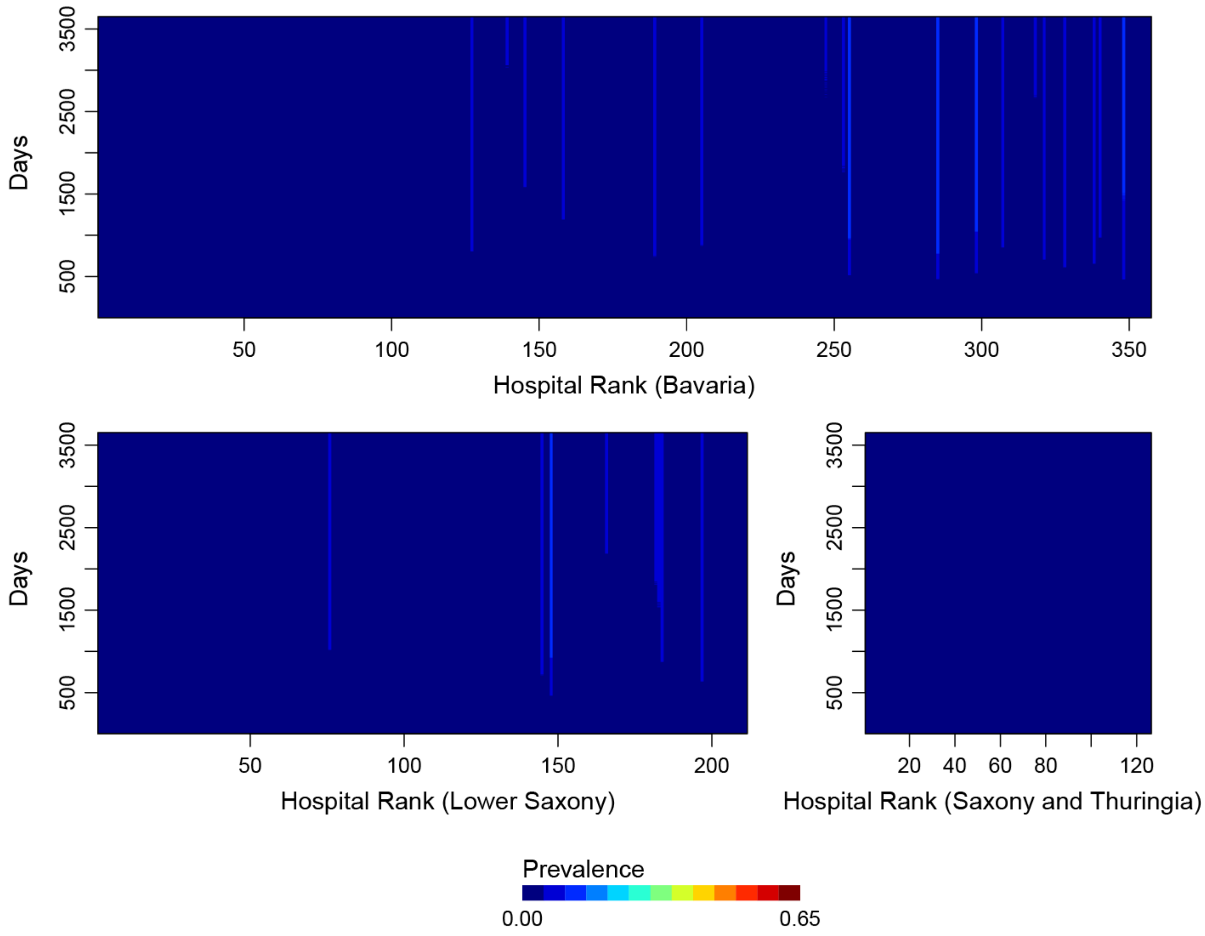

Figure S15. Daily prevalence at admission in single community nodes. The x-axis represents hospitals, ranked from largest to smallest (left to right) based on the average number of hospital beds that were occupied there per day.

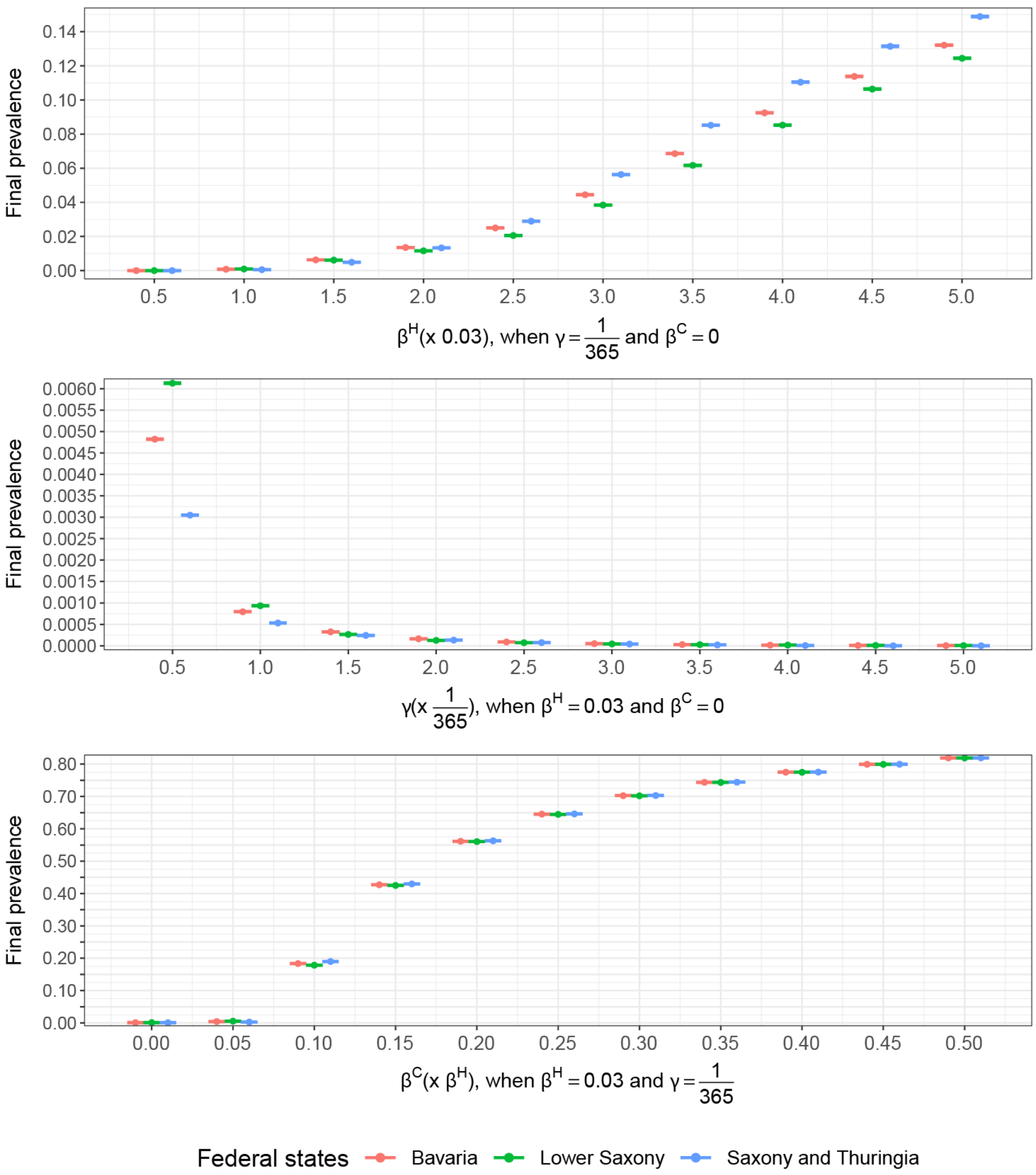

Figure S16. Final prevalence in community nodes for varying transmission parameters in the studied federal states. The upper and lower bars indicate the spread between the largest and lowest prevalence in the weekly patterns. When there is no variation, the bars converge to a single line. β^H and β^C are the transmission rates in hospitals and in community nodes, respectively. γ is the colonization clearing rate.
